# A Review of Ultrathin Piezoelectric Films

**DOI:** 10.3390/ma16083107

**Published:** 2023-04-14

**Authors:** Bingyue Li, Zude Xie, Hanzhong Liu, Liming Tang, Keqiu Chen

**Affiliations:** School of Physics and Electronics, Hunan University, Changsha 410082, China

**Keywords:** ultrathin films, energy harvesting, piezoelectric mechanism, in-plane piezoelectricity, out-of-plane piezoelectricity

## Abstract

Due to their high electromechanical coupling and energy density properties, ultrathin piezoelectric films have recently been intensively studied as key materials for the construction of miniaturized energy transducers, and in this paper we summarize the research progress. At the nanoscale, even a few atomic layers, ultrathin piezoelectric films have prominent shape anisotropic polarization, that is, in-plane polarization and out-of-plane polarization. In this review, we first introduce the in-plane and out-of-plane polarization mechanism, and then summarize the main ultrathin piezoelectric films studied at present. Secondly, we take perovskite, transition metal dichalcogenides, and Janus layers as examples to elaborate the existing scientific and engineering problems in the research of polarization, and their possible solutions. Finally, the application prospect of ultrathin piezoelectric films in miniaturized energy converters is summarized.

## 1. Introduction

Since the discovery and synthesis of piezoelectric materials, researchers have gradually realized that piezoelectric technology can be widely used in biomedicine [1,2,3], flexible electronic devices [4,5], flexible generators [6,7,8,9], ultrathin film vibrators [10], piezoelectric photoelectronic solar cells [11,12] and many other fields. Many kinds of piezoelectric materials have been reported to have high piezoelectric properties and high electromechanical coupling, such as epitaxial wurtzite Sc_x_Al_1-x_N ultrathin films [13] and nanocomposite [14,15]. In addition, the premise of high performance, coupled with the principle of being green, environmentally friendly, safe and sustainable, calls for the emergence of a number of new materials; for instance, lead-free 0.5Ba(Zr_0.2_Ti_0.8_)O_3_–0.5(Ba_0.7_Ca_0.3_)TiO_3_ nanowire [16], lead-free perovskite [17] and lead-free nanocomposites [18].

**Figure 1:**
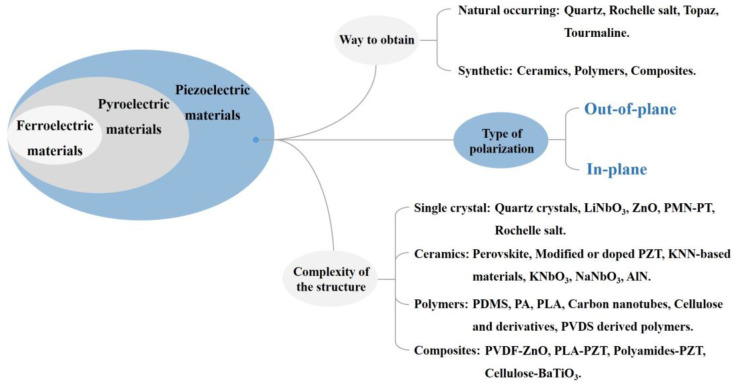
The relationship between piezoelectric materials, pyroelectric materials and ferroelectric materials. Piezoelectric materials are classified according to three different criteria.

Generally speaking, microscopically, potential piezoelectric materials only originate from materials with a non-centrosymmetric microstructure. The work of Gowoon Cheon et al. [19] describes 325 potential two-dimensional (2D) piezoelectric materials. They are not piezoelectric in the bulk phase but lack central symmetry in the monolayer. There are 32 crystal symmetry groups, among which 21 have broken central symmetry. Surprisingly, 20 of them have directly observed piezoelectric responses. Piezoelectric materials were first discovered in naturally occurring natural products (quartz crystals and Rochelle salt). Monocrystalline, polycrystalline and polymer materials with better piezoelectric properties were discovered or synthesized later [20]. The classification of piezoelectric materials is given in Figure 1. Grounding three different criteria, completely different classification results will be showed. Furthermore, each type of material uses several materials to illustrate [21,22]. Most importantly, our focus is on all the parts of the diagram marked blue, that is to say, the type of polarization is a key factor. In Section 3, in-plane and out-of-plane polarization will be introduced in more detail.

Nevertheless, previous attention and research has focused mainly on materials with broken central symmetry [23,24,25]. Recently, in the reporting of D.-S. Park et al. [26], the piezoelectric phenomenon was realized in a centrosymmetric oxide. Unlike other methods, this exciting breakthrough was achieved by regulating the inserted oxygen vacancy with an applied electric field. In this regard, it also provides a new way to induce piezoelectricity. Under the action of a direct current (DC) electric field, the degree of defect migration has been significantly improved, which is represented by the numerical leap of dielectric constant. As a result, the achievement leads to the ultra-high voltage electrical property of the film [27]. Xiaoqing Yang et al. also used the first-principles method to predict the longitudinal piezoelectric effect of the oxide family AO (A = Cd, Ba, Sr, Ca, Mg, etc.) under the action of biaxial strain [28]. After this, it was also proved experimentally that the piezoelectric constant is enhanced in the 2D limit [29]. At the macro scale, a strain with a particular large gradient is required to cause significant polarization in the material, whereas, at the nano scale, even a small strain can do so [30,31]. For example, for the star material MoS_2_ in the 2D monolayer, an independent monolayer material is obtained experimentally through various preparation methods, and its piezoelectric properties are characterized [32,33]. These works provide an experimental basis and proof for 2D ultrathin piezoelectricity. Additionally, the layer odd–even dependence of the piezoelectric response was also found [33].

Aiming to achieve more adjustable piezoelectricity, a new fabrication method of traditional 2D materials [34] has been developed. At the same time, large piezoelectric responses have been achieved in the Janus monolayer through first-principles calculations [35]. Typically, the effect of inducing vertical dipoles by breaking the symmetry of the out-of-plane structure caused this huge response [36]. Of course, this mechanism can also be applied to group-III chalcogenide monolayers, such as Ga_2_SSe, with both inversion breaking and specular symmetry [37], though they still remain thermodynamically stable. Very recently, with the continuous progress of scientific and technological means, Bohayra Mortazavi et al. realized the first-principles study of piezoelectricity on MA_2_Z_4_ (M = Cr, Mo, W; A = Si, Ge; Z = N, P) monolayer through machine learning [38]. It is enough to raise concerns about new types of ultrathin films. The unique excellent performance of this group can even have a certain competitiveness in various application fields (nanoelectronics, optoelectronics and energy conversion nanosystems) [39].

In this review, we first elaborate the basic working principle of the piezoelectric effect in Section 2, and discuss the piezoelectric properties of thin film materials. Additionally, we also observe that the intrinsic difference of polarization mechanism between thin film and bulk material lies in the difference of anisotropy. Afterwards, we find that there are layer thickness effects and odd–even effects in these materials that already have theoretical and experimental foundations. Hence, we also summarize the physical mechanism grounding the instance of MoS_2_ and hexagonal boron nitride (h-BN). Notably, the modes applied by the strain have the potential to induce novel quantum effects, which will be the focus of future research. In Section 3, we classify ultrathin piezoelectric films from an entirely different perspective from most of the previous reviews: ultrathin in-plane piezoelectric films and ultrathin out-of-plane piezoelectric films. Moreover, based on transition metal dichalcogenides (TMDs), Janus structure and novel ultrathin films, the piezoelectric polarization mechanism and modification methods are analyzed. For the purpose of helping the workers who are interested in ultrathin piezoelectric films to clarify the components and their respective characteristics, we compiled the details included in this review. Finally, in Section 4, we summarize the current research progress of ultrathin piezoelectric films and their applications in energy harvesting and conversion and point out that there are still unavoidable defects and limitations in the fabrication of 2D materials within the scope of current technology.

## 2. Piezoelectric Effect and Polarization Mechanisms

The piezoelectric effect results from the uneven distribution of ions in the crystal structure in some materials [40]. “Electric charge that accumulates in response to applied mechanical stress in materials that have non-centrosymmetric crystal structures” is defined as piezoelectricity [41]. A piezoelectric material is one that can achieve energy collection and conversion through the piezoelectric effect. Typically, the dipoles are randomly distributed in the crystal structure and call for an equilibrium state of electroneutrality on a macroscopic level. In these materials, though, electrons and holes move in opposite directions towards the metal-semiconductor interface due to a piezoelectric potential, which is generated by an external stimulus (polarization). The behavior by which the dipole tends to the same random direction is known as the piezoelectric polarization effect [42]. The connection between elasticity and electrical behavior is made successfully from then.

**Figure 2:**
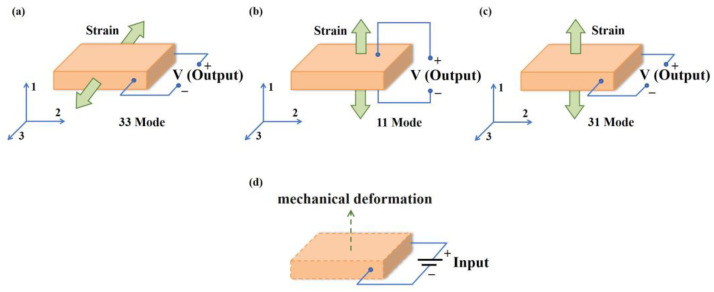
The schematic diagram of the piezoelectric energy conversion and collection devices (direct piezoelectric effect) and piezoelectric actuators (inverse piezoelectric effect). (**a**) 33 mode; (**b**) 11 mode; (**c**) 31 mode of direct piezoelectric effect. (**d**) Inverse piezoelectric effect.

The piezoelectric effect can be modulated by regulating the interface properties of the electron transport as well as photoelectric properties [43]. The piezoelectric polarization effect can be divided into direct effect and inverse effect. The schematic diagram of the two working mechanisms is shown in Figure 2. Direct piezoelectric effects occur when the existing dipole balance in the crystal structure is disturbed due to the application of mechanical stress (stretching or compression) or vibrations. This disturbance results in an unbalanced distribution of charge, contributing to a surface charge density that is able to be captured by the electrode [44]. In this process, mechanical energy is converted to electric energy [45]. Conversely, the conversion of electrical energy into mechanical energy is an inverse piezoelectric effect. The whole process can be described as applying an electric field of a certain magnitude to the piezoelectric material. This effect causes a mechanical displacement within the material. The intrinsic equation of the two effects can be expressed as bellow [41]:

(1) Direct Effect: D=dT+εE

(2) Inverse Effect: S=sT+dE

where T stands for the stress, d stands for the piezoelectric constant, S stands for the strain, D stands for the electric displacement, E stands for the electric field intensity, s stands for the mechanical flexibility, and ε stands for the dielectric constant of the material. The four piezoelectric coefficients $d_{ij}$, $e_{ij}$, $g_{ij}$, $h_{ij}$ are expressed as [46]:

(3) $$\left\{\begin{array}{c} d_{ij}={\left(\frac{\partial D_{i}}{\partial T_{j}}\right)}^{E}={\left(\frac{\partial S_{j}}{\partial E_{i}}\right)}^{T}\\ e_{ij}={\left(\frac{\partial D_{i}}{\partial S_{j}}\right)}^{E}={-\left(\frac{\partial T_{j}}{\partial E_{i}}\right)}^{S}\\ g_{ij}={-\left(\frac{\partial E_{i}}{\partial T_{j}}\right)}^{D}={\left(\frac{\partial S_{j}}{\partial D_{i}}\right)}^{T}\\ h_{ij}=-{\left(\frac{\partial E_{i}}{\partial S_{j}}\right)}^{D}=-{\left(\frac{\partial T_{j}}{\partial D_{i}}\right)}^{S} \end{array}\right.$$

these two terms after the equal sign correspond to direct and inverse piezoelectric effects, respectively.

Taking 2D TMDs as an example, the 2H phase structure has the symmetry of $D_{3h}$ space group. The in-plane and out-of-plane piezoelectric coefficients $d_{11}$, $d_{31}$ and the elastic stiffness coefficients $c_{11}$, $c_{31}$ satisfy the following relation [47]:

(4) $$d_{11}=\frac{e_{11}}{C_{11}-C_{12}},\;d_{31}=\frac{e_{31}}{C_{11}+C_{12}}.$$

With regard to 2D materials with $C_{2v}$ space group symmetry, this relationship becomes [48]:

(5) $$d_{11}=\frac{e_{11}C_{22}-e_{12}C_{12}}{C_{11}C_{22}-{C_{12}}^{2}},\;d_{12}=\frac{e_{12}C_{11}-e_{11}C_{12}}{C_{11}C_{22}-{C_{12}}^{2}}.$$

Piezoelectric energy conversion and collection devices usually work with a direct piezoelectric effect, and the types are mainly the 33, 11 and 31 modes; these are shown in Figure 2a–c. In the 33 and 11 modes, the applied stress is in the same direction as the generated voltage, while in the 31 mode the applied stress is axial, and the voltage is vertical. The piezoelectric output is under the control of the operation mode. The 33 mode has excellent voltage output, while a superior manifestation aspect to high current output is found in the 31 mode [49,50]. On the other hand, the inverse piezoelectric effect is put into use in piezoelectric actuators, as shown in Figure 2d.

In various polarization mechanisms [51,52], it is common that the layer thickness effect and odd–even effect are reported [53]. As a matter of fact, they are attributed to changes in interlayer coupling and symmetry. Even layers samples have inversion symmetry, while odd layers samples destroy that. For example, direct and inverse piezoelectric effects have been experimentally confirmed in a single and few layers of MoS_2_. Nevertheless, in-plane piezoelectricity only exists in odd layers, and decreases rapidly with increasing number of layers. The reason for the phenomenon is that the response of the opposite direction layers is cancelled out. Meanwhile, any strain or electric field perpendicular to its surface will theoretically produce a zero piezoelectric response [30].

## 3. Piezoelectric Thin Films

The single layer of piezoelectric film is prepared experimentally, and the piezoelectricity constant is enhanced in the 2D limit [29,32,33]. These results and data mean the nano-scale ultrathin piezoelectric films are not only limited to theoretical calculation, but also signal a new era in the field of piezoelectric research. Table 1 and Table 2 show the piezoelectric coefficients of 2D piezoelectric film measured under the inverse piezoelectric effect and the direct piezoelectric effect, respectively. We find that several of these materials, such as ZnO, MoS_2_ and Zr_2_P_2_BrCl, show strong anisotropic piezoelectric polarization effects. This property is useful in that can be used to achieve different energy conversion effects by applying stretching or compression effects in different directions. In order to screen, distinguish and store different energy signals, it is often necessary to use anisotropic materials to design devices.

### 3.1. Ultrathin In-Plane Piezoelectric Films

**Figure 3:**
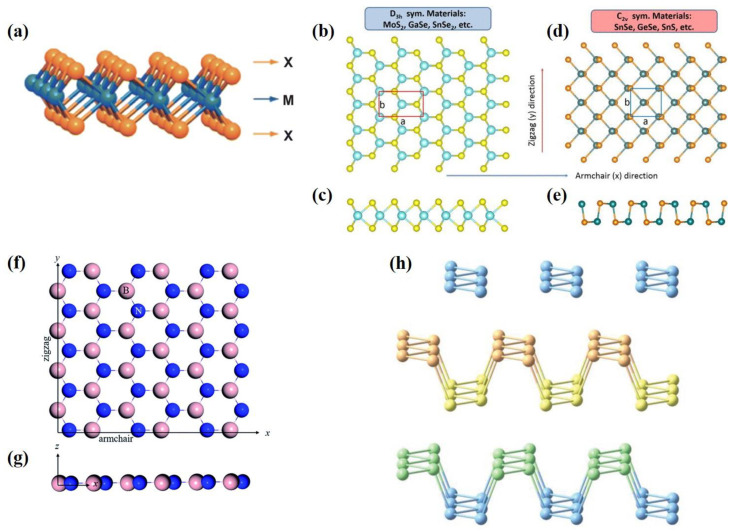
The schematic diagram of the ultrathin in-plane films [42,68,101,102]. (**a**) transition metal dichalcogenides (TMDs) (M = transition metal atoms; X = dichalcogenide atoms). Reproduced with permission from [42]. Copyright 2020, Springer Nature. (**b**) Top view of $D_{3h}$ symmetry materials. Reprinted from [68], with the permission of AIP Publishing. (**c**) Side view of $D_{3h}$ symmetry materials. Reprinted from [68], with the permission of AIP Publishing. (**d**) Top view of $C_{2v}$ symmetry materials. Reprinted from [68], with the permission of AIP Publishing. (**e**) Side view of $C_{2v}$ symmetry materials. Reprinted from [68], with the permission of AIP Publishing. (**f**) Top view of hexagonal boron nitride (h-BN). Reprinted with permission from [101]. Copyright 2018 Royal Society of Chemistry. (**g**) Side view of h-BN. Reprinted with permission from [101]. Copyright 2018 Royal Society of Chemistry. (**h**) Crystalline views of black phosphorene. The P atoms in different positions are marked with different colors. Used with permission of John Wiley & Sons—Books, from [102]. Copyright 2020 WILEY—V C H VERLAG GMBH & CO. KGAA.

With the development of energy equipment towards miniaturization, the critical scale of transducers needs to be reduced. The scale of ultrathin films in this review is defined to the nanometer scale. Most 2D materials have intrinsic piezoelectric polarization. We introduce the piezoelectric properties of TMDs, h-BN and black phosphorus (BP). A schematic of each of these structures is shown in Figure 3.

**Figure 4:**
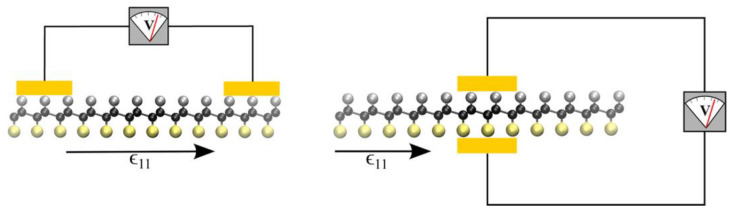
Hydrogen and fluorine adsorbed on the different side of graphene induced both in-plane and out-of-plane piezoelectric effects [107]. Reprinted with permission from [107]. Copyright 2013 American Chemical Society.

Ultrathin piezoelectric films are expected to be widely used as a result of their stress-electric conversion characteristics. Graphene has the longest history of study among 2D materials [103,104,105], and therefore was the material in which researchers initially tried to achieve the piezoelectric effect. Grounding the piezoelectric mechanism, the effect calls for the breaking of central symmetry. Swapnil Chandratre and Pradeep Sharma achieved the piezoelectric effect in 2D materials for the first time by digging triangular holes in graphene [106]. Shortly after, Mitchell T. Ong et al. chemically modified graphene with hydrogen and fluorine adsorbed on different position, and induced both in-plane and out-of-plane piezoelectric effects [107], as shown in Figure 4.

**Figure 5:**
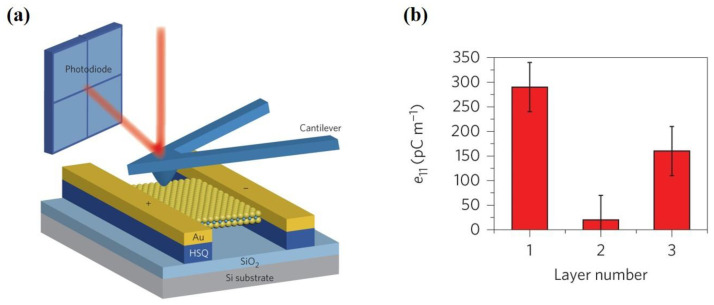
(**a**) The schematic diagram of a device for measuring the in-plane piezoelectric stress. Two hydrogen silsesquioxane (HSQ) columns are under the MoS_2_ film, which are on a SiO_2_/Si substrate with an Au electrode on the upper side. The AFM probe changes the fold degree of the film and deflects the laser beam, so that the stress can be obtained through the load change on the cantilever beam [33]. Reproduced with permission from [33]. Copyright 2014, Springer Nature. (**b**) The measured piezoelectric response of MoS_2_ is odd–even dependent as the number of layers increases [33]. Reproduced with permission from [33]. Copyright 2014, Springer Nature.

**Figure 6:**
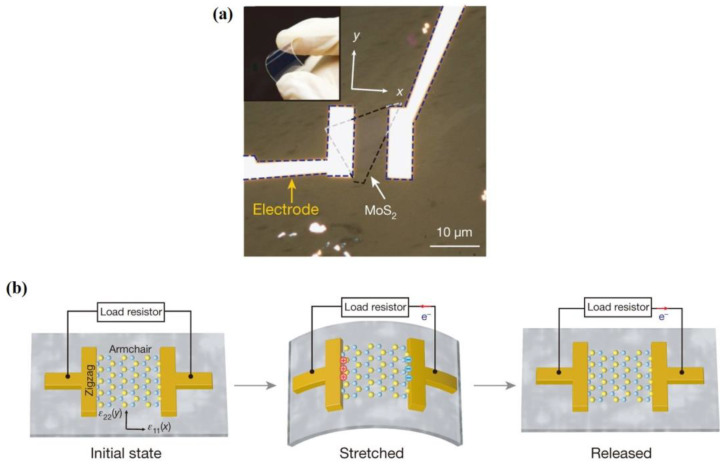
(**a**) Flexible electronic devices based on single layer MoS_2_ thin films [71]. Reproduced with permission from [71]. Copyright 2014, Springer Nature. (**b**) The operating principle of this flexible electronic device. When it is stretched and compressed, piezoelectric polarized charges of opposite sign are induced, making it possible to detect piezoelectric output in the external circuit [71]. Reproduced with permission from [71]. Copyright 2014, Springer Nature.

**Figure 7:**
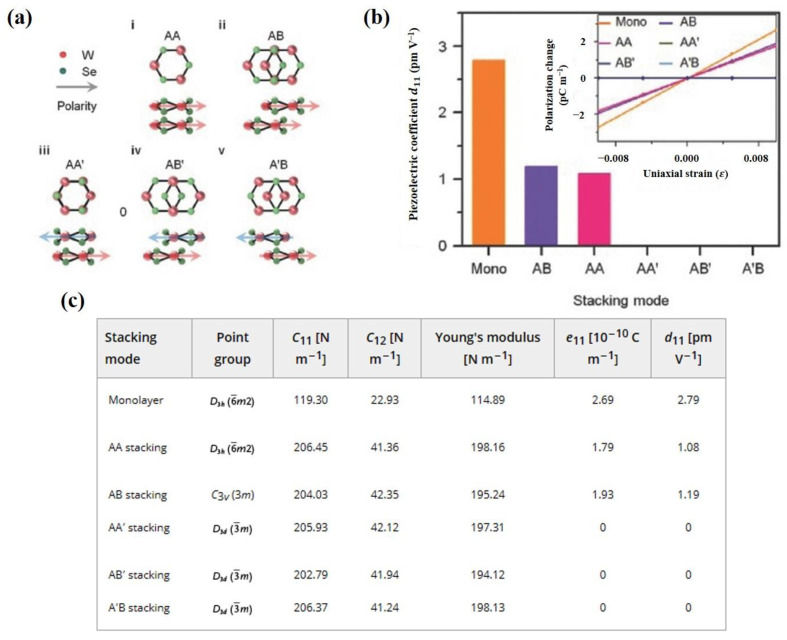
(**a**) Stacking structure for bilayer-WSe_2_ [72]. Signs i–v correspond to AA, AB, AA’, AB’ and A’B stacking. Used with permission of John Wiley & Sons—Books, from [72]. Copyright 2017 WILEY—V C H VERLAG GMBH & CO. KGAA. (**b**) Simulated piezoelectric coefficient (d_11_) of monolayer WSe_2_ and stacked WSe_2_ [72]. Used with permission of John Wiley & Sons—Books, from [72]. Copyright 2017 WILEY—V C H VERLAG GMBH & CO. KGAA. (**c**) DFT calculation results based on different stacking of WSe_2_ [72]. Used with permission of John Wiley & Sons—Books, from [72]. Copyright 2017 WILEY—V C H VERLAG GMBH & CO. KGAA.

Ultrathin nanosheets of layered TMDs have tunable electronic structures, so as to be attractive for piezoelectric energy harvesting. TMDs can be represented as MX_2_ (M = Cr, Mo, W, Nb, Ta and X = S, Se, Te) [64]. Monolayer TMDs are constructed by a metal plane surrounded by two dichalcogenide planes [108]. Hexagonal structured monolayer TMDs are usually semiconductors and belong to $D_{3h}$ space group, the breaking of whose symmetry leads to piezoelectricity [109]. Duerloo et al. first predicted that 2D TMD materials are piezoelectric by the first-principles method [66]. MoS_2_ is the most widely studied TMD and it is worth going into more detail on. Monolayer MoS_2_ generates intrinsic electric dipoles due to the displacement of cationic Mo atoms and anionic S atoms when subjected to external strains. Additionally, the inherent electric dipoles lead to a voltage between the two sides of MoS_2_. The piezoelectric property of monolayer MoS_2_ is only restricted to the in-plane ($d_{11}$) direction rather than the out-of-plane ($d_{33}$) direction, since the symmetry feature along the vertical z-axis of monolayer MoS_2_ stays the same as in the initial state. Furthermore, Wu [71] and Zhu [33] et al. experimentally realized piezoelectricity in monolayer MoS_2_. Zhu et al. measured a piezoelectric coefficient of $e_{11}$ = 2.9 × 10^–10^ C m^−1^ in a free-standing single layer of MoS_2_. Additionally, they observed a finite and zero piezoelectric response in odd and even numbers of layers, respectively, which is in sharp contrast to bulk piezoelectric materials [33]. This is because the intrinsic difference of the polarization mechanism between thin film and bulk material relies on the difference of anisotropy. The measurement results are shown in Figure 5b. This is because symmetry is broken only in odd layers, while it is restored in even layers. The measurement method is shown in Figure 5a. The MoS_2_ film was indented with a scanning atomic force microscopy (AFM) probe, aiming to convert the in-plane stress to an out-of-plane force. The induced stress then changed the load on the tip and the curvature of the cantilever, which could be measured by the deflection of a laser beam [33]. Additionally, in the study of Wu et al., as shown in Figure 6, MoS_2_ was placed on a polyethylene terephthalate (PET) flexible substrate [71]. Uniaxial strain was applied to the MoS_2_ in the manner of mechanically bending the substrate. Therefore, periodic stretching and releasing of the substrate can generate piezoelectric outputs in external circuits with alternating polarity [71]. However, in principle, the exfoliated monolayer MoS_2_ lacks mechanical durability. Ju-Hyuck Lee et al. fabricated bilayer WSe_2_ with a mechanical durability of up to 0.95% of strain via turbostratic stacking [Figure 7a]. This form can increase the degrees of freedom in the bilayer symmetry and finally lead to non-centrosymmetry in the bilayers [Figure 7b] [72,110]. The density function theory (DFT) simulation results are shown in Figure 7c.

There has been a boom in research about in-plane piezoelectric 2D materials. Yang et al. calculated the piezoelectric coefficient of monolayer MX (M = Sn or Ge, X = Se or S) and found that the in-plane piezoelectric coefficient d_11_ of MX was the biggest among 2D materials [68]. This result is two orders of magnitude larger than that of routinely used piezoelectric semiconductors, such as ZnO and monolayer MoS_2_. This is because monolayer group IV monochalcogenides have a unique “puckered” $C_{2v}$ symmetry, as well as electronic structure.

**Figure 8:**
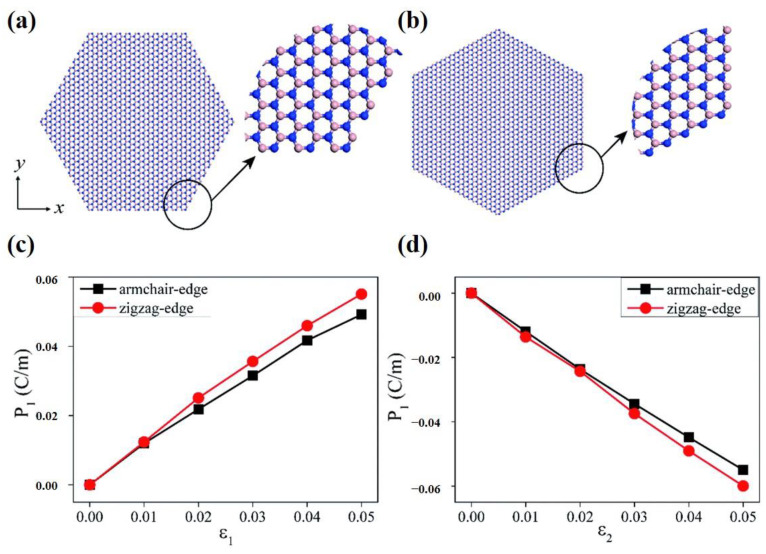
(**a**,**b**) show the atomic structures of armchair and zigzag edges of a hexagonal shaped boron nitride nanosheets structure, respectively [101]. Reprinted with permission from [101]. Copyright 2018 Royal Society of Chemistry. (**c**,**d**) show the polarization changes of hexagonal shape boron nitride nanosheets structures along the x- and y-directions, respectively, as a result of stretching [101]. Reprinted with permission from [101]. Copyright 2018 Royal Society of Chemistry.

**Figure 9:**
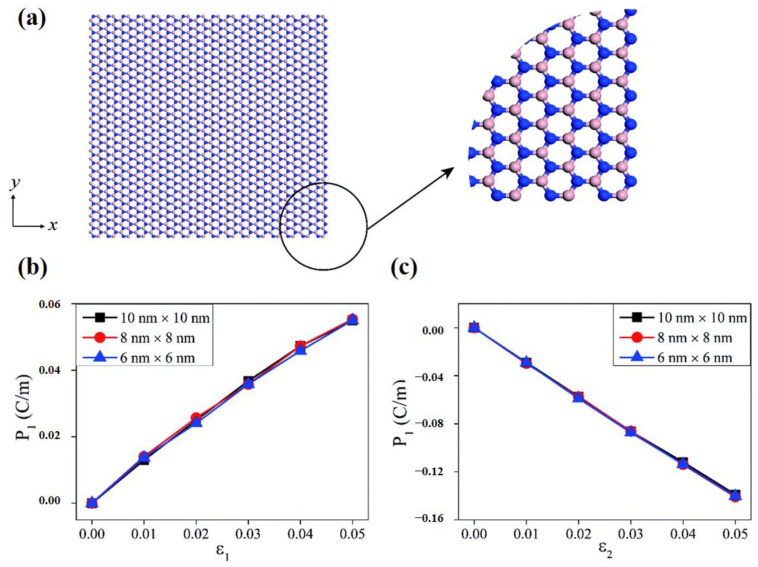
(**a**) The atomic structure of rectangular shaped boron nitride nanosheets. The amplified area shows armchair and zigzag boundaries along the x and y directions, respectively [101]. Reprinted with permission from [101]. Copyright 2018 Royal Society of Chemistry. (**b**) The polarization changes of different size boron nitride nanosheets when strained along the armchair direction and (**c**) zigzag direction [101]. Reprinted with permission from [101]. Copyright 2018 Royal Society of Chemistry.

Monolayer h-BN is a wide-band insulator with the same crystal structure as graphene. Unlike graphene, monolayer h-BN is piezoelectric due to the asymmetry introduced into the structure by the two sublattices (B and N). The elastic and piezoelectric effects were theoretically calculated using Born’s long-wavelength theory [111]. In 2020, Yang Nan et al. studied hexagonal boron nitride nanosheets through molecular dynamics simulations. Their simulation results indicated that the piezoelectric constants of boron nitride nanosheets depend very strongly on the macroscopic shape (Figure 8), while being nearly independent of the macroscopic size (Figure 9) [101]. In addition, when strain is applied to h-BN, some novel quantum effects, such as the quantum spin Hall phase [112], are born. These novel quantum effects, when combined with piezoelectric polarization effects, are bound to prompt researchers to ponder more interesting scientific questions.

Black phosphorus (BP) is a layered material that can be obtained by mechanical stripping. The piezoelectric properties of BP are demonstrated due to its non-centrosymmetric crystal structure [113]. In addition, surface-oxidization is an effective way to enhance the piezoelectricity of black phosphorene, since this method can break the structural symmetry. By first-principles methods, the calculated piezoelectric coefficients $d_{11}$ for surface-oxidized BP are manifested as 88.54 pm V^−1^, which is comparable to those of group-IV monochalcogenides and larger than that of 2D h-BN and MoS_2_ [77]. However, this method of stripping or traditional chemical and physical vapor deposition can likely introduce defects or other impurities, which can interfere with the results of the study. It is worth further consideration by researchers.

**Figure 10:**
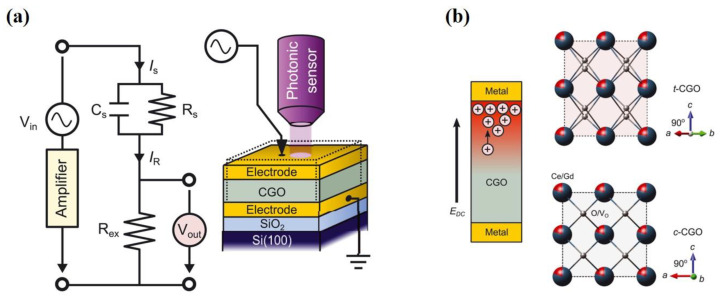
(**a**) Schematics of the experimental setup. The thicknesses of CeO_2–x_ (CGO) films are in the range of ~1.25 to ~1.8 mm [26]. From [26]. Reprinted with permission from AAAS. (**b**) The phase transition of CGO from cubic to tetragonal phase under an EDC [26]. From [26]. Reprinted with permission from AAAS.

There are many kinds of means to control the properties of 2D materials, such as substitutional doping, staking, surface adatoms, or defects to break the centrosymmetry of pristine materials [114]. However, these strategies of regulating mainly affect the out-of-plane piezoelectric properties, which will be discussed later. The in-plane piezoelectric properties are little affected by these methods in principle, since the intrinsic asymmetry of materials is the factor most important to in-plane piezoelectric properties. Unconventionally, in 2022, Park et al. induced high levels of piezoelectricity in centrosymmetric oxides [26]. They used a DC electric field to rearrange the oxygen vacancies in the CeO_2–x_ (CGO) thin film. The crystallographic symmetry was broken and piezoelectric effects were induced in centrosymmetric materials. The piezoelectricity is measured by applying an additional alternating current (AC) electric field (E_AC_). When the AC electric field is in the same direction as DC, oxygen vacancies are pushed up, which causes the material to expand. Conversely, when the AC electric field is in the opposite direction to DC, oxygen vacancies are pushed oppositely [27]. Their results show that the piezoelectric coefficients ($d_{33}$) can reach up to nearly 200,000 pm V^−1^ at a frequency of 10 mHz under a DC electric field of 1 MV cm^−1^. This data is particularly significant, being two orders of magnitude larger than the ferroelectric oxide. They also discovered that the field-induced redistribution of oxygen vacancies could induce a cubic-to-tetragonal structural transition [26], as shown in Figure 10b. This inspired people to apply this method to more materials or look for new mechanisms to generate piezoelectricity.

### 3.2. Ultrathin Out-of-Plane Piezoelectric Films

**Figure 11:**
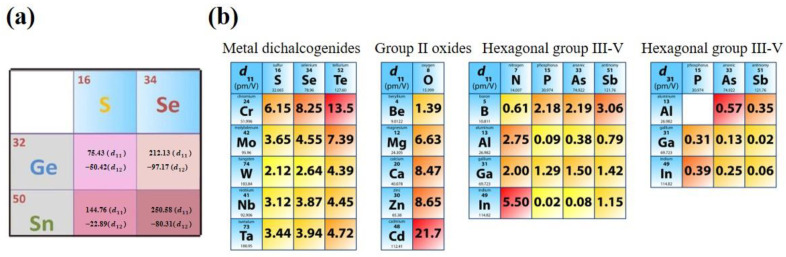
(**a**,**b**) The piezoelectric coefficients of some common two-dimensional (2D) materials [54]. Reproduced with permission from [54]. Copyright 2018, Springer Nature.

With the systematic study of piezoelectricity in 2D materials, a variety of correlated novel piezoelectric devices have been successively fabricated in the field of energy harvesting, actuators, strain-tuned electronics, and optoelectronics. Most 2D materials, as shown in Figure 11a,b, have only in-plane piezoelectricity, which limits their applications in vertically integrated nanoelectromechanical systems [64]. Due to the limitation of external conditions and structural stability, it is difficult to make advanced functional devices with piezoelectric materials. Herein, Table 3 shows that the piezoelectric properties of common materials in different polarization directions are experimentally confirmed [54,115]. If 2D materials can obtain large out-of-plane piezoelectricity, they will be more widely used in a variety of piezoelectric applications. It has stimulated more and more research on innovative regulatory means, such as doping, construction of Janus structures and so on, because of the puzzle of how to obtain out-of-plane polarization.

#### 3.2.1. Out-of-Plane Piezoelectricity Obtained by Controlling Structures

Out-of-plane piezoelectricity is usually obtained by controlling the crystal structure of the 2D materials, such as through defect engineering, deformation or doping. TMDs are ideal candidates as low-dimensional piezoelectric materials, owing to their structural non-centrosymmetry [33]. Due to in-plane inversion symmetry breaking, it was proved through experimental characterization and theoretical calculation that only intrinsic in-plane piezoelectricity exists in TMDs [64,66,117]. From this, breaking inversion symmetry in an out-of-plane direction is an efficient method to realize the out-of-plane piezoelectric polarization [115]. It is noted that the layered TMDs have the natural advantage of being established into out-of-plane piezoelectric samples through crystal structure transformation.

##### Deformation

**Figure 12:**
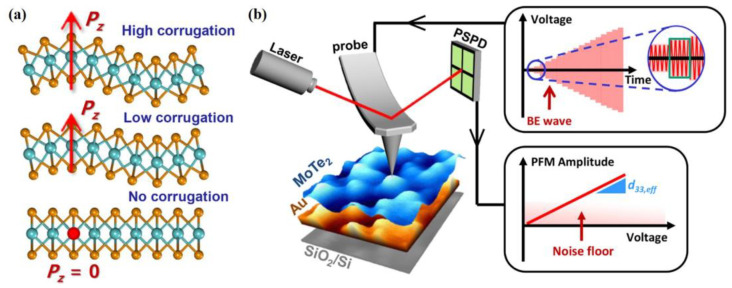
(**a**) The different degrees of corrugation engineering [118]. Reprinted with permission from [118]. Copyright 2018 American Chemical Society. (**b**) The deformation of MoTe_2_ film driven by Au fold was observed by atomic force microscopy (AFM). Waveform diagram of band excitation (BE) with Vac. The relationship between the amplitude of piezoelectric response force microscope (PFM) and the magnitude of alternating current (AC) voltage [118]. Reprinted with permission from [118]. Copyright 2018 American Chemical Society.

While only in-plane piezoelectricity exists on the crystal symmetry, it is also possible to generate out-of-plane piezoelectricity by way of bringing strain to the gradient. The schematic diagram of different degrees of corrugation is shown in Figure 12a. As long as the 2D TMDs deform along the out-of-plane direction, the internal flexoelectricity polarization occurs along this axial direction. In this sense, the out-of-plane piezoelectricity of 2H phase MoTe_2_ can be modulated by either controlling the roughness of the substrate or the thickness of MoTe_2_ sample [118], as shown in Figure 12b.

##### Defect Engineering

Studies to date have demonstrated the possibility of generating an out-of-plane piezoelectric property in TMDs through defect engineering [114]. Still, it is a challenge to effectively modulate surface corrugation due to the limited modulation of substrate roughness or thickness. Thus comes the question of whether flexoelectricity can be generated at atomic scales or not. If so, out-of-plane piezoelectricity will be generated regardless of the roughness or thickness. The surface vacancy of a Te atom is generated by the thermal annealing process, which contributes to greater surface curvature and thus produces greater out-of-plane piezoelectricity [119].

Furthermore, out-of-plane piezoelectricity in TMDs with layered structure can be formed by using ion beams to engineer the defects. For instance, the formation of Te defects caused by helium ion beam irradiation on the multilayer MoTe_2_ actually leads to the out-of-plane piezoelectricity [120].

##### Doping

Chemical doping is also a new choice for obtaining out-of-plane piezoelectricity. Growing the material on the substrate can achieve this aim experimentally. The growth of graphene on SiO_2_ substrate causes the chemical interaction between graphene and SiO_2_ surface, thus realizing periodic doping. This interaction can induce band gap opening and dipole moment polarization of the graphene layer. Hence, it grants the material out-of-plane piezoelectricity [55]. In electronics manufacturing and energy harvesting applications, it is possible to use piezoelectric effects along the vertical direction in 2D TMDs.

#### 3.2.2. Out-of-Plane Piezoelectricity in Janus Structures

##### Conventional TMDs Janus Structures

**Figure 13:**
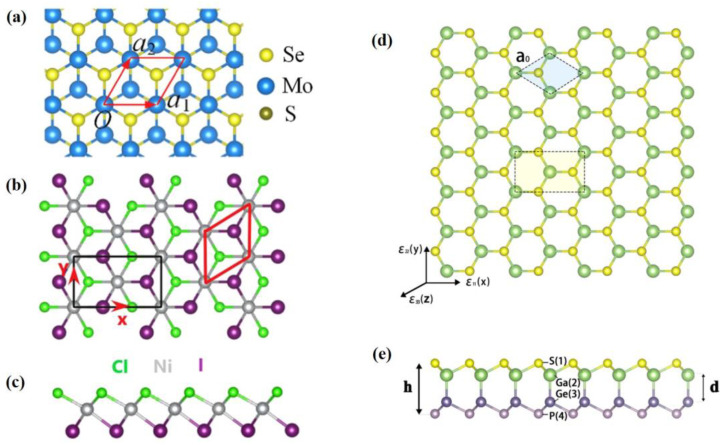
The schematic diagram of the ultrathin out-of-plane films [121,122,123]. (**a**) Top view of MoSSe. Reprinted from [122], copyright 2019, with permission from Elsevier. (**b**) Top view of NiClI. Reprinted from [121], with the permission of AIP Publishing. (**c**) Side view of NiClI. Reprinted from [121], with the permission of AIP Publishing. (**d**) Top view of the GeP–GaS monolayer. Reprinted from [123], copyright 2020, with permission from Elsevier. (**e**) Side view of the GeP–GaS monolayer. Reprinted from [123], copyright 2020, with permission from Elsevier.

Another atomic-scale approach has been proposed to induce out-of-plane piezoelectricity through constructing asymmetric TMD monolayers, known as conventional Janus structures. In addition to the most common MoSSe [65], NiXY (X/Y = Cl, Br, I; X ≠ Y) is also an example. The structure diagram is shown in Figure 13a–c. In this regard, NiXY is a novel piezoelectric ferromagnetic material with these two unique properties. These Janus monolayer materials, such as NiClBr and NiBrI, have strong in-plane as well as out-of-plane piezoelectricity. It is found that NiClI is a ferrovalley material with magnetic anisotropy, which is an ideal material for ultrathin piezoelectric devices [121].

##### Janus Derivatives

As a matter of fact, Janus can not only be based on TMDs, but also be built in typical single-layer structures with hexagonal primitive cells or a buckled honeycomb monolayer. A series of Janus derivatives are obtained by substituting atoms (GeP–GaS, SiP–AlS, and SnP–InS). The structure diagram is shown in Figure 13d,e. This type of structure lacks mirror symmetry, leading to a large out-of-plane piezoelectricity [123]. Single-layer SiN–GaS also exhibits considerable out-of-plane piezoelectricity [124]. Janus M_2_SeX (M = Ge, Sn; X = S, Te) have both large in-plane piezoelectric coefficients $d_{11}$ (up to 345.08 pm/V) and out-of-plane piezoelectric coefficients $d_{31}$ (up to 3.83 pm/V) [125]. As well as the larger surface piezoelectricity, Janus derivatives can also combine a variety of properties to offer opportunities for the manufacture of multifunctional devices. The research of the Janus structure of X_2_PAs monolayers shows that the out-of-plane piezoelectricity and flexible properties play an important part in improving the performance of multifunctional sensing and controlling of nanodevices [126]. The piezoelectric ferromagnetism material Fe_2_IX (X = Cl and Br) combines piezoelectricity, topological properties and ferromagnetism orders. In this sense, it provides a potential platform for multi-functional spintronic devices with a large gap and high T_C_ (429 K) [127].

Janus materials also have tribo-piezoelectricity. The sliding of the in-plane interlayer leads to a remarkable enhancement in the vertical piezoelectricity aspect. In the process of Janus bilayer sliding, the tribological energy will convert into electrical energy. Hence, it brings a new perspective to creating new piezoelectric nanogenerators [122]. The construction of a Janus structure can fully demonstrate the modification of traditional 2D ultrathin films.

#### 3.2.3. Out-of-Plane Piezoelectricity in Multi-Element Transition Metal Materials

**Figure 14:**
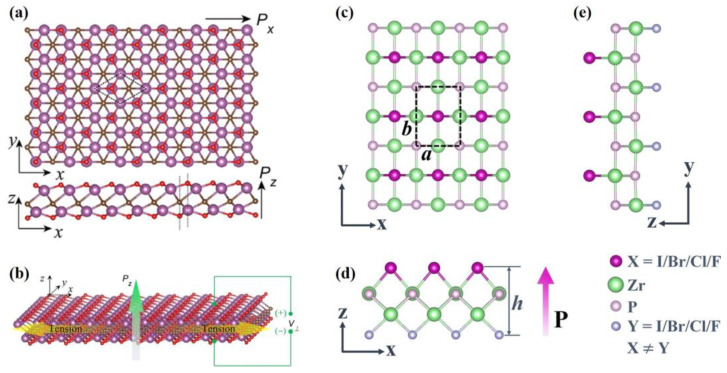
The schematic diagram of the ultrathin out-of-plane films [86,89]. (**a**) Top and side view of Sc_2_CO_2_ MXene. Reprinted from [86], copyright 2019, with permission from Elsevier. (**b**) A Sc_2_CO_2_ MXene piezoelectric cantilever. Reprinted from [86], copyright 2019, with permission from Elsevier. (**c**) Top view of the Zr_2_P_2_BrCl monolayer. Reprinted from [89], copyright 2023, with permission from Elsevier. (**d**,**e**) Side view of the Zr_2_P_2_BrCl monolayer from x-z plane and z-y plane. Reprinted from [89], copyright 2023, with permission from Elsevier.

If simple Janus can achieve structure asymmetry, can more complex structures do the same? The answer is, of course, yes. In 2D materials, a quantity of three- and four-membered compound monolayers are non-centrosymmetric, such as MXenes, lithium-based ternary chalcogenides and so on. A schematic of each of these structures is shown in Figure 14. Due to the uneven charge distribution caused by non-mirror symmetry, these multi-element transition metal materials have strong out of plane piezoelectricity.

Strikingly, M_2_CO_2_ (M = Sc, Y, La) has an in-plane piezoelectricity comparable to 2H-MoS_2_ as well as extraordinary out-of-plane piezoelectricity. The piezoelectric coefficient $d_{31}$ of Sc_2_CO_2_ MXene reaches 0.78 pm V^−1^. At 2.5% strain, 0.1 V vertical piezoelectric voltage can only be produced in such ultrathin Sc_2_CO_2_ monolayer [86]. Similarly, BiCrX_3_(X = S, Se, and Te) is a kind of piezoelectric ferromagnetism material with both in-plane and out-of-plane polarization at room temperature. Additionally, this is another breakthrough in high-performance piezoelectric materials [128].

**Figure 15:**
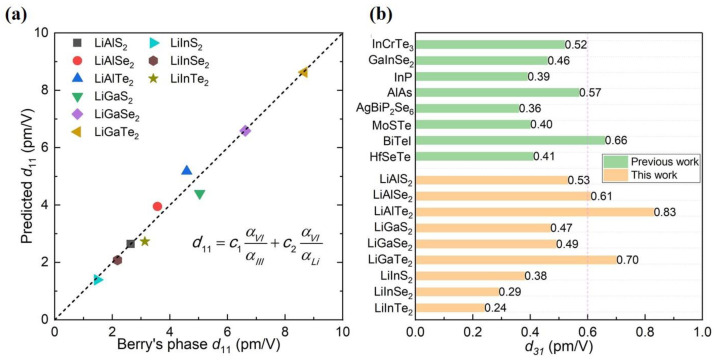
(**a**) Relationship between piezoelectric coefficient d_11_ and polarizability of LiMX_2_ monolayer [87]. Reprinted from [87], copyright 2021, with permission from Elsevier. (**b**) Comparison of the piezoelectric coefficient $d_{31}$ of LiMX_2_ single layer with reported piezoelectric materials [87]. Reprinted from [87], copyright 2021, with permission from Elsevier.

The out-of-plane piezoelectric coefficients $d_{31}$ of 2D monolayer Li-based ternary chalcogenides LiMX_2_ (M = Al, Ga, In; X = S, Se, Te), such as LiAlSe_2_, LiGaTe_2_ and LiAlTe_2_, reach up to 0.61, 0.70 and 0.83 pm/V, respectively. This is owing to the unique double-buckled stacking structure of these LiMX_2_ monolayers [87]. Later, it was found that γ phase structure (γ-LialS_2_ and γ-LialSe_2_) also has an excellent out-of-plane piezoelectric coefficient; even the number is twice as high as that of β phase structure [88]. When compared with the piezoelectric coefficients of other materials as shown in Figure 15, it is evident that the γ-LiMX_2_ structures are very promising materials for high-performance piezoelectric nanodevices.

Zr_2_P_2_BrCl monolayer is of particular interest for its ferroelasticity, controllable anisotropic properties along the in-plane direction and outstanding out-of-plane piezoelectricity. These monolayers have a strong out-of-plane piezoelectricity since the heterogeneous charge distribution results from its broken mirror symmetry. The piezoelectric coefficient $d_{33}$ = 129.705 pm V^−1^ of Zr_2_P_2_BrCl monolayer is actually significant, which is two orders of magnitude higher than MoSTe multilayers. It can even be a new alternate material for the design of memory devices, robot bionic skin or multipurpose nanodevices [89].

**Figure 16:**
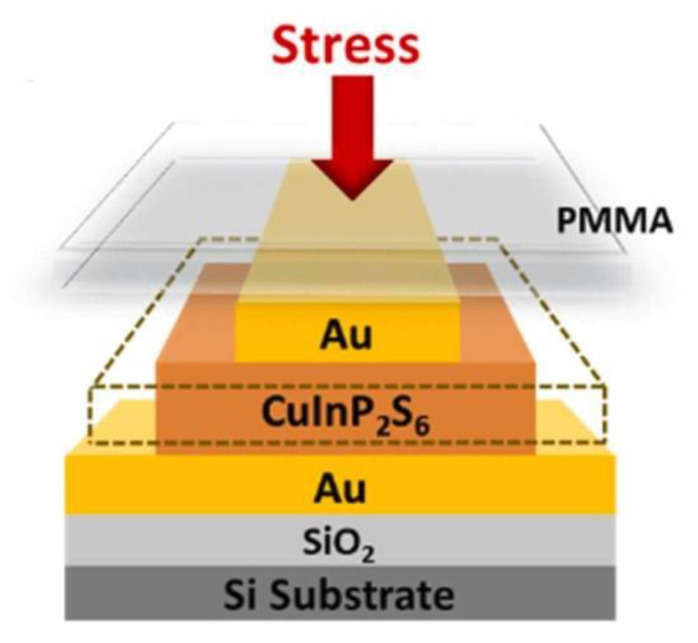
The schematic diagram of piezoelectric device based on CuInP_2_S_6_ (CIPS) [63]. Reprinted from [63], copyright 2022, with permission from Elsevier.

In addition to the materials mentioned above, the low-layer CuInP_2_S_6_ (CIPS) nanosheet also exhibits great performance. CIPS is a ferroelectric material with a high $d_{33}$ piezoelectric coefficient of 17.4 pm V^−1^. The data is superior to that of other reported 2D piezoelectric films to date. The intense out-of-plane piezoelectricity in the ultrathin CIPS contributes to the integration of nanoscale energy transducers, and thus eventually the production of nanogenerators [63]. A CIPS-based piezotronics device under compressive stress is shown in Figure 16.

## 4. Concluding Remarks

The intrinsic differences of ultrathin films, thick films and bulk materials regarding polarization due to gradually reduced anisotropy are explored in the first part of this review. When the thickness of material gradually decreases to several atoms thick, the effective piezoelectric polarization will enhance significantly. Secondly, several promising materials with strong anisotropy, such as ZnO, MoS_2_ and Zr_2_P_2_BrCl monolayer, are screened by summarizing the piezoelectric coefficient, polarization direction and preparation quality of the ultrathin films. It is noted that there are great difficulties in the acquisition and preparation of ultrathin piezoelectric films. The available methods have inevitable shortcomings and limitations, such as introducing a large number of defects, which is also a great challenge faced by the large family of 2D materials. Thirdly, based on the traditional piezoelectric hexagonal boron nitride structure, the effect of layer thickness, strain modes and sizes on polarization, and the novel quantum effects induced by it, such as topological state, quantum Hall effect, etc., are analyzed in detail. Particularly, the polarization mechanism of a few-atom-thick material is demonstrated by taking the symmetrically polarized monolayer TMDs as an example. In addition, to illustrate the construction and modification of a 2D piezoelectric material module, the Janus structure is taken as an example. Through this review, we have found that ultrathin piezoelectric films have made rapid progress and become a promising structure in the development of miniaturized energy conversion equipment.

## Figures and Tables

**Table 1:** Piezoelectric coefficient of common piezoelectric materials (pm V^−1^).

Materials	$d_{11}$ (pm V^−1^)	$d_{12}$ (pm V^−1^)	$d_{33}$ (pm V^−1^)	$d_{31}$ (pm V^−1^)	Ref
Graphene/SiO_2_			14		[54,55]
Graphene oxide				0.24	[56]
Graphene (K doped)				0.23	[57]
Graphene (Li doped)				0.15	[57]
Graphene (H doped)				0.11	[57]
Graphene (F doped)				0.0018	[57]
Graphene (F, Li doped)				0.30	[57]
Graphene (H, F doped)				0.03	[57]
BaTiO_3_			8		[58]
PZT			7–15		[59]
PVDF			−36		[60]
C_3_N_4_			1		[61]
LiNbO_3_			7.50		[30]
GaPO_4_			~8.50		[62]
CuInP_2_S_6_ (3.5 nm)			17.40		[63]
CuInP_2_S_6_ (82 nm)			83		[63]
2H-CrS_2_	6.15				[64]
2H-CrSe_2_	8.25				[64]
2H-CrTe_2_	13.45				[64]
2H-MoS_2_	3.65				[64,65]
	2.5–4				[54,66]
	3.73				[66,67,68,69]
	3.34				[65]
	3.73				[65]
			1.35		[30]
			1.03		[70]
	3				[66,71]
2H-MoSe_2_	4.55				[64,65]
	4.17				[65]
	4.72				[65,66]
2H-MoTe_2_	7.39				[64,65]
	7.00				[65]
	9.13				[65,66]
2H-WS_2_	2.12				[64,65]
	2.02				[65]
	2.19				[65,66]
2H-WSe_2_	2.64				[64,65]
	2.53				[65]
	2.79				[65,66]
	~3.26				[72]
2H-WTe_2_	4.39				[64,65]
	4.29				[65]
	4.60				[65,66]
2H-NbS_2_	3.12				[64]
2H-NbSe_2_	3.87				[64]
2H-NbTe_2_	4.45				[64]
2H-TaS_2_	3.44				[64]
2H-TaSe_2_	3.94				[64]
2H-TaTe_2_	4.72				[64]
BeO	1.39				[64]
MgO	6.63				[64]
CaO	8.47				[64]
ZnO	8.65		14.30–26.70		[64,73]
			23.70		[74]
			21.50		[75]
CdO	21.70				[64]
PbO	73.10		29.60	3.90	[64,76]
α-SbAs	243.45	−63.65			[69]
α-SbP	142.44	−27.64			[69]
α-SbN	118.29	−13.20			[69]
α-AsP	18.90	−4.74			[69]
α-AsN	29.14	−5.75			[69]
α-PN	6.94	−2.44			[69]
β-SbAs	1.65			−0.03	[69]
β-SbP	2.26			−0.03	[69]
β-SbN	5.30			−0.26	[69]
β-AsP	0.67			0.01	[69]
β-AsN	4.83			−0.09	[69]
β-PN	2.77			−0.09	[69]
BP	2.18				[64]
surface-oxidized BP	88.54				[77]
BAs	2.19				[64]
BSb	3.06				[64]
AlN	2.75				[64]
AlP	0.09				[64]
AlAs	0.38			0.57	[42,64]
AlSb	0.79			0.35	[42,64]
GaN	2.00				[64]
GaP	1.29			0.31	[42,64]
GaAs	1.50			0.13	[42,64]
GaSb	1.42			0.02	[42,64]
InN	5.50				[64]
InP	0.02			0.39	[42,64]
InAs	0.08			0.25	[42,64]
InSb	1.15			0.06	[42,64]
BN	0.61				[64]
h-BN	0.60				[47,66,67]
CdS			32.80		[78]
GaS	1.72				[37]
	2.06				[47,76]
GaSe	1.77				[37]
	2.30				[47,68,76]
GaTe	1.93				[37]
InS	1.12				[37]
InSe	1.98				[37]
	1.46				[47,76]
InTe	1.18				[37]
GeS	75.43	−50.42			[42,68]
A-GeS	20.71	11.16			[48]
H-GeS	−5.65	5.65			[48]
GeSe	212.13	−97.17			[42,68,69]
A-GeSe	40.61	14.96			[48]
H-GeSe	−4.88	4.88			[48]
SnS	144.76	−22.89	~1.85		[42,68,79]
A-SnS	91.56	−4.02			[48]
H-SnS	−5.28	5.28			[48]
SnSe	250.58	−80.31			[42,68,69]
A-SnSe	74.73	0.85			[48]
H-SnSe	−4.63	4.63			[48]
SnS_2_			2.20		[80]
			~5		[81]
α-In_2_Se_3_			0.34		[82]
			0.53		[63]
In_2_Te_3_	10.64			0.40	[83]
Ga_2_SSe	5.23			0.07	[37,76]
Ga_2_STe	2.46			0.25	[37,76]
Ga_2_SeTe	2.32			0.21	[37,76]
In_2_SSe	8.47			0.18	[37,76]
In_2_STe	1.91			0.25	[37,76]
In_2_SeTe	4.73			0.13	[37,76]
GaInS_2_	8.33			0.38	[37,76]
GaInSe_2_	3.19			0.46	[37,76]
GaInTe_2_	2.99			0.32	[37,76]
InCrTe_3_	6.94			0.52	[83]
1H-WSO	1.8				[35]
1H-WSeO	1.9				[35]
1H-WTeO	2.8				[35]
MoSSe	3.76		0.10	0.02	[36,54,65,76]
MoSeTe	5.30			0.03	[65,76]
MoSTe	5.04			0.03	[65,76]
WSSe	2.26			0.01	[65,76]
WSeTe	3.52			0.01	[65,76]
WSTe	3.33			0.01	[65,76]
ZrSSe				0.01	[84]
ZrSeTe				−0.19	[84]
ZrSTe				0.004	[84]
HfSSe				0.05	[84]
HfSeTe				0.41	[84]
HfSTe				0.18	[84]
BiTeI	8.49			0.56	[85]
Sc_2_CO_2_				0.78	[86]
Y_2_CO_2_				0.40	[86]
La_2_CO_2_				0.65	[86]
LiAlS_2_	2.64			0.53	[87,88]
γ-LiAlS_2_	8.04			1.17	[88]
LiAlSe_2_	3.57			0.61	[87,88]
γ-LiAlSe_2_	10.50			1.62	[88]
LiAlTe_2_	4.58			0.83	[87,88]
γ-LiAlTe_2_	11.30			0.39	[88]
LiGaS_2_	5.03			0.47	[87,88]
γ-LiGaS_2_	12.02			0.05	[88]
LiGaSe_2_	6.60			0.49	[87,88]
γ-LiGaSe_2_	14.48			0.31	[88]
LiGaTe_2_	8.66			0.70	[87,88]
γ-LiGaTe_2_	17.44			0.84	[88]
LiInS_2_	1.48			0.38	[87,88]
γ-LiInS_2_	5.90			0.12	[88]
LiInSe_2_	2.18			0.29	[87,88]
γ-LiInSe_2_	7.81			0.28	[88]
LiInTe_2_	3.13			0.24	[87,88]
γ-LiInTe_2_	8.93			0.83	[88]
Zr_2_P_2_IBr			29.95	0.93	[89]
Zr_2_P_2_ICl			91.12	2.65	[89]
Zr_2_P_2_IF			33.87	0.43	[89]
Zr_2_P_2_BrCl			129.71	3.21	[89]
Zr_2_P_2_BrF			22.77	0.09	[89]
Zr_2_P_2_ClF			51.05	0.91	[89]

**Table 2:** Piezoelectric coefficient of common piezoelectric materials (pC N^−1^).

Materials	$d_{11}$ (pC N^−1^)	$d_{12}$ (pC N^−1^)	$d_{33}$ (pC N^−1^)	$d_{31}$ (pC N^−1^)	Ref
BaTiO_3_			191	78	[90]
			~190		[90,91]
ZnO			5.90		[90]
			12		[92]
			12.40		[93]
			~5–10		[90,94]
Quartz	2.30				[90]
ST-cut Quartz	2.30				[92]
PMN-PT			~2000–3000		[90]
PMN-PZT/PT			~1500–2000		[95,96]
PZT			~60–130		[90]
			225–590	110	[41,44,93]
			289–380		[92]
			117		[92]
			~250–700		[97]
AlN			4.50		[92]
			6.40		[92]
			5		[98,99]
Sapphire			6.40		[92]
GaN			4.50		[92]
128° cut LiNbO_3_			12		[92]
36° YX cut LiTaO_3_			12		[92]
PVDF Film			−33	23	[44]
			−35		[92]
			~20–30		[90,100]

**Table 3:** Relevant information of the experimentally confirmed piezoelectric 2D materials [54]. Reproduced with permission from [54]. Copyright 2018, Springer Nature.

2D Materials	Crystal Structure	Piezoelectric Direction	Estimated Piezocoefficient	Notes
Monolayer MoS_2_	Hexagonal	In-plane Angle dependence	$d_{11}=2.5-4\;pm/V$ [66] $e_{11}=250-400\;pC/m$ [33,66]	Odd–even effect with the thickness increased
Monolayer h-BN	Hexagonal	In-plane Angle dependence	$e_{11}=100-400\;pC/m$ [66]	Odd–even effect with the thickness increased [53]
Graphitic carbon nitride		In-plane	$e_{11}=218\;pC/m$ [61]	Existence of piezoelectricity regardless of thickness
Doped graphene	Hexagonal	Out-of-plane	$d_{33}=1.4\;nm/V$ [55]	Extrinsic piezoelectricity
α-In_2_Se_3_	Rhombohedral	Out-of-plane and in-plane		Indirectly confirmed by the ferroelectricity [116]
Janus MoSSe	Hexagonal	Out-of-plane and in-plane	$d_{33}=0.1\;pm/V$ [36]	

## Data Availability

No new data were created or analyzed in this study.

## References

[1] Surmenev R.A., Chernozem R.V., Pariy I.O., Surmeneva M.A. (2021). A review on piezo- and pyroelectric responses of flexible nano- and micropatterned polymer surfaces for biomedical sensing and energy harvesting applications. Nano Energy.

[2] Laurila M.-M., Peltokangas M., Montero K.L., Verho J., Haapala M., Oksala N., Vehkaoja A., Mäntysalo M. (2022). Self-powered, high sensitivity printed e-tattoo sensor for unobtrusive arterial pulse wave monitoring. Nano Energy.

[3] Wu C.-W., Ren X., Xie G., Zhou W.-X., Zhang G., Chen K.-Q. (2022). Enhanced High-Temperature Thermoelectric Performance by Strain Engineering in BiOCl. Phys. Rev. Appl..

[4] Mariello M., Fachechi L., Guido F., De Vittorio M. (2021). Conformal, Ultra-thin Skin-Contact-Actuated Hybrid Piezo/Triboelectric Wearable Sensor Based on AlN and Parylene-Encapsulated Elastomeric Blend. Adv. Funct. Mater..

[5] Li W., Yang T., Liu C., Huang Y., Chen C., Pan H., Xie G., Tai H., Jiang Y., Wu Y. (2022). Optimizing Piezoelectric Nanocomposites by High-Throughput Phase-Field Simulation and Machine Learning. Adv. Sci..

[6] Lu L., Ding W., Liu J., Yang B. (2020). Flexible PVDF based piezoelectric nanogenerators. Nano Energy.

[7] Shepelin N.A., Glushenkov A.M., Lussini V.C., Fox P.J., Dicinoski G.W., Shapter J.G., Ellis A.V. (2019). New developments in composites, copolymer technologies and processing techniques for flexible fluoropolymer piezoelectric generators for efficient energy harvesting. Energy Environ. Sci..

[8] Yu D., Zheng Z., Liu J., Xiao H., Huangfu G., Guo Y. (2021). Superflexible and Lead-Free Piezoelectric Nanogenerator as a Highly Sensitive Self-Powered Sensor for Human Motion Monitoring. Nano-Micro Lett..

[9] Zhong J., Zhong Q., Zang X., Wu N., Li W., Chu Y., Lin L. (2017). Flexible PET/EVA-based piezoelectret generator for energy harvesting in harsh environments. Nano Energy.

[10] Takeshita T., Nguyen T.-V., Zymelka D., Takei Y., Kobayashi T. (2022). Mechanical characteristics of laminated film vibrator using an ultra-thin MEMS actuator. J. Micromech. Microeng..

[11] Michael G., Hu G., Zheng D., Zhang Y. (2019). Piezo-phototronic solar cell based on 2D monochalcogenides materials. J. Phys. D Appl. Phys..

[12] Wu W., Wang Z.L. (2016). Piezotronics and piezo-phototronics for adaptive electronics and optoelectronics. Nat. Rev. Mater..

[13] Casamento J., Chang C.S., Shao Y.-T., Wright J., Muller D.A., Xing H., Jena D. (2020). Structural and piezoelectric properties of ultra-thin Sc_x_Al_1−x_N films grown on GaN by molecular beam epitaxy. Appl. Phys. Lett..

[14] Yu S., Zhang Y., Yu Z., Zheng J., Wang Y., Zhou H. (2021). PANI/PVDF-TrFE porous aerogel bulk piezoelectric and triboelectric hybrid nanogenerator based on in-situ doping and liquid nitrogen quenching. Nano Energy.

[15] Shi K., Chai B., Zou H., Shen P., Sun B., Jiang P., Shi Z., Huang X. (2021). Interface induced performance enhancement in flexible BaTiO_3_/PVDF-TrFE based piezoelectric nanogenerators. Nano Energy.

[16] Zhou Z., Bowland C.C., Malakooti M.H., Tang H., Sodano H.A. (2016). Lead-free 0.5Ba(Zr_0.2_Ti_0.8_)O_3_–0.5(Ba_0.7_Ca_0.3_)TiO_3_ nanowires for energy harvesting. Nanoscale.

[17] Zheng T., Wu J., Xiao D., Zhu J. (2018). Recent development in lead-free perovskite piezoelectric bulk materials. Prog. Mater. Sci..

[18] Jeong C.K., Park K.-I., Ryu J., Hwang G.-T., Lee K.J. (2014). Large-Area and Flexible Lead-Free Nanocomposite Generator Using Alkaline Niobate Particles and Metal Nanorod Filler. Adv. Funct. Mater..

[19] Cheon G., Duerloo K.-A.N., Sendek A.D., Porter C., Chen Y., Reed E.J. (2017). Data Mining for New Two- and One-Dimensional Weakly Bonded Solids and Lattice-Commensurate Heterostructures. Nano Lett..

[20] Mohith S., Upadhya A.R., Navin K.P., Kulkarni S.M., Rao M. (2020). Recent trends in piezoelectric actuators for precision motion and their applications: A review. Smart Mater. Struct..

[21] Wang J., Cao X.-H., Zeng Y.-J., Luo N.-N., Tang L.-M., Chen K.-Q. (2023). Excellent thermoelectric properties of monolayer MoS2-MoSe2 aperiodic superlattices. Appl. Surf. Sci..

[22] Jia P.-Z., Xie Z.-X., Deng Y.-X., Zhang Y., Tang L.-M., Zhou W.-X., Chen K.-Q. (2022). High thermoelectric performance induced by strong anharmonic effects in monolayer (PbX)_2_ (X = S, Se, Te). Appl. Phys. Lett..

[23] He R., Wang D., Luo N., Zeng J., Chen K.-Q., Tang L.-M. (2023). Nonrelativistic Spin-Momentum Coupling in Antiferromagnetic Twisted Bilayers. Phys. Rev. Lett..

[24] Yuan Z.-L., Sun Y., Wang D., Chen K.-Q., Tang L.-M. (2021). A review of ultra-thin ferroelectric films. J. Phys. Condens. Matter.

[25] Li Q., Chen K.-Q., Tang L.-M. (2020). Large Valley Splitting in van der Waals Heterostructures with Type-III Band Alignment. Phys. Rev. Appl..

[26] Park D.-S., Hadad M., Riemer L.M., Ignatans R., Spirito D., Esposito V., Tileli V., Gauquelin N., Chezganov D., Jannis D. (2022). Induced giant piezoelectricity in centrosymmetric oxides. Science.

[27] Li F. (2022). Breaking symmetry for piezoelectricity. Science.

[28] Yang X., Liu C., Fang L., Su T., Hu M., Chen Y., Zhou B., Su H., Kong X., Bellaiche L. (2022). Strain-induced ferroelectricity and piezoelectricity in centrosymmetric binary oxides. Phys. Rev. B.

[29] Wu T., Zhang H. (2015). Piezoelectricity in Two-Dimensional Materials. Angew. Chem. Int. Ed..

[30] Brennan C.J., Ghosh R., Koul K., Banerjee S.K., Lu N., Yu E.T. (2017). Out-of-Plane Electromechanical Response of Monolayer Molybdenum Disulfide Measured by Piezoresponse Force Microscopy. Nano Lett..

[31] Cao X.-H., Wu D., Zeng J., Luo N.-N., Zhou W.-X., Tang L.-M., Chen K.-Q. (2021). Controllable anisotropic thermoelectric properties in 2D covalent organic radical frameworks. Appl. Phys. Lett..

[32] Kim S.K., Bhatia R., Kim T.-H., Seol D., Kim J.H., Kim H., Seung W., Kim Y., Lee Y.H., Kim S.-W. (2016). Directional dependent piezoelectric effect in CVD grown monolayer MoS 2 for flexible piezoelectric nanogenerators. Nano Energy.

[33] Zhu H., Wang Y., Xiao J., Liu M., Xiong S., Wong Z.J., Ye Z., Ye Y., Yin X., Zhang X. (2015). Observation of piezoelectricity in free-standing monolayer MoS_2_. Nat. Nanotechnol..

[34] Liu D., Zeng J., Jiang X., Tang L., Chen K. (2023). Exact first-principles calculation reveals universal moiré potential in twisted two-dimensional materials. Phys. Rev. B.

[35] Varjovi M.J., Yagmurcukardes M., Peeters F.M., Durgun E. (2021). Janus two-dimensional transition metal dichalcogenide oxides: First-principles investigation of WXO monolayers with X = S, Se, and Te. Phys. Rev. B.

[36] Lu A.-Y., Zhu H., Xiao J., Chuu C.-P., Han Y., Chiu M.-H., Cheng C.-C., Yang C.-W., Wei K.-H., Yang Y.Y.P. (2017). Janus monolayers of transition metal dichalcogenides. Nat. Nanotechnol..

[37] Guo Y., Zhou S., Bai Y., Zhao J. (2017). Enhanced piezoelectric effect in Janus group-III chalcogenide monolayers. Appl. Phys. Lett..

[38] Mortazavi B., Javvaji B., Shojaei F., Rabczuk T., Shapeev A.V., Zhuang X. (2020). Exceptional piezoelectricity, high thermal conductivity and stiffness and promising photocatalysis in two-dimensional MoSi2N4 family confirmed by first-principles. Nano Energy.

[39] Safaei M., Sodano H.A., Anton S.R. (2019). A review of energy harvesting using piezoelectric materials: State-of-the-art a decade later (2008–2018). Smart Mater. Struct..

[40] Das Mahapatra S., Mohapatra P.C., Aria A.I., Christie G., Mishra Y.K., Hofmann S., Thakur V.K. (2021). Piezoelectric Materials for Energy Harvesting and Sensing Applications: Roadmap for Future Smart Materials. Adv. Sci..

[41] Covaci C., Gontean A. (2020). Piezoelectric Energy Harvesting Solutions: A Review. Sensors.

[42] Li F., Shen T., Wang C., Zhang Y., Qi J., Zhang H. (2020). Recent Advances in Strain-Induced Piezoelectric and Piezoresistive Effect-Engineered 2D Semiconductors for Adaptive Electronics and Optoelectronics. Nano-Micro Lett..

[43] Lin P., Yan X., Li F., Du J., Meng J., Zhang Y. (2017). Polarity-Dependent Piezotronic Effect and Controllable Transport Modulation of ZnO with Multifield Coupled Interface Engineering. Adv. Mater. Interfaces.

[44] Mishra S., Unnikrishnan L., Nayak S.K., Mohanty S. (2019). Advances in Piezoelectric Polymer Composites for Energy Harvesting Applications: A Systematic Review. Macromol. Mater. Eng..

[45] Zeng Y.-J., Liu Y.-Y., Pan H., Ding Z.-K., Zhou W.-X., Tang L.-M., Li B., Chen K.-Q. (2022). Thermoelectric Conversion From Interface Thermophoresis and Piezoelectric Effects. Front. Phys..

[46] (1988). IEEE Standard on Piezoelectricity.

[47] Li W., Li J. (2015). Piezoelectricity in two-dimensional group-III monochalcogenides. Nano Res..

[48] Hu T., Dong J. (2016). Two new phases of monolayer group-IV monochalcogenides and their piezoelectric properties. Phys. Chem. Chem. Phys..

[49] Sezer N., Koç M. (2020). A comprehensive review on the state-of-the-art of piezoelectric energy harvesting. Nano Energy.

[50] Liu H., Zhong J., Lee C., Lee S.-W., Lin L. (2018). A comprehensive review on piezoelectric energy harvesting technology: Materials, mechanisms, and applications. Appl. Phys. Rev..

[51] Pan H., Tang L.-M., Chen K.-Q. (2022). Quantum mechanical modeling of magnon-phonon scattering heat transport across three-dimensional ferromagnetic/nonmagnetic interfaces. Phys. Rev. B.

[52] Zheng Y., Jiang X., Xue X.-X., Yao X., Zeng J., Chen K.-Q., Wang E., Feng Y. (2022). Nuclear Quantum Effects on the Charge-Density Wave Transition in NbX_2_ (X = S, Se). Nano Lett..

[53] Li Y., Rao Y., Mak K.F., You Y., Wang S., Dean C.R., Heinz T.F. (2013). Probing Symmetry Properties of Few-Layer MoS_2_ and h-BN by Optical Second-Harmonic Generation. Nano Lett..

[54] Cui C., Xue F., Hu W.-J., Li L.-J. (2018). Two-dimensional materials with piezoelectric and ferroelectric functionalities. Npj 2D Mater. Appl..

[55] Rodrigues G.D.C., Zelenovskiy P., Romanyuk K., Luchkin S., Kopelevich Y., Kholkin A. (2015). Strong piezoelectricity in single-layer graphene deposited on SiO_2_ grating substrates. Nat. Commun..

[56] Chang Z., Yan W., Shang J., Liu J.Z. (2014). Piezoelectric properties of graphene oxide: A first-principles computational study. Appl. Phys. Lett..

[57] Ong M.T., Reed E.J. (2012). Engineered Piezoelectricity in Graphene. ACS Nano.

[58] Baturin A.S., Bulakh K., Zenkevich A.V., Minnekaev M.N., Chuprik A.A. (2012). Study of the ferroelectric properties of BaTiO3 films grown on an iron sublayer using atomic force microscopy. J. Surf. Investig. X-ray, Synchrotron Neutron Tech..

[59] Nagarajan V., Junquera J., He J.Q., Jia C.L., Waser R., Lee K., Kim Y.K., Baik S., Zhao T., Ramesh R. (2006). Scaling of structure and electrical properties in ultrathin epitaxial ferroelectric heterostructures. J. Appl. Phys..

[60] Hussain N., Zhang M.-H., Zhang Q., Zhou Z., Xu X., Murtaza M., Zhang R., Wei H., Ou G., Wang D. (2019). Large Piezoelectric Strain in Sub-10 Nanometer Two-Dimensional Polyvinylidene Fluoride Nanoflakes. ACS Nano.

[61] Zelisko M., Hanlumyuang Y., Yang S., Liu Y., Lei C., Li J., Ajayan P.M., Sharma P. (2014). Anomalous piezoelectricity in two-dimensional graphene nitride nanosheets. Nat. Commun..

[62] Syed N., Zavabeti A., Ou J.Z., Mohiuddin M., Pillai N., Carey B.J., Zhang B.Y., Datta R.S., Jannat A., Haque F. (2018). Printing two-dimensional gallium phosphate out of liquid metal. Nat. Commun..

[63] Io W.F., Wong M.-C., Pang S.-Y., Zhao Y., Ding R., Guo F., Hao J. (2022). Strong piezoelectric response in layered CuInP2S6 nanosheets for piezoelectric nanogenerators. Nano Energy.

[64] Blonsky M.N., Zhuang H.L., Singh A.K., Hennig R.G. (2015). Ab Initio Prediction of Piezoelectricity in Two-Dimensional Materials. ACS Nano.

[65] Dong L., Lou J., Shenoy V.B. (2017). Large In-Plane and Vertical Piezoelectricity in Janus Transition Metal Dichalchogenides. ACS Nano.

[66] Duerloo K.-A.N., Ong M.T., Reed E.J. (2012). Intrinsic Piezoelectricity in Two-Dimensional Materials. J. Phys. Chem. Lett..

[67] Feng W., Guo G.-Y., Yao Y. (2016). Tunable magneto-optical effects in hole-doped group-IIIA metal-monochalcogenide monolayers. 2D Mater..

[68] Fei R., Li W., Li J., Yang L. (2015). Giant piezoelectricity of monolayer group IV monochalcogenides: SnSe, SnS, GeSe, and GeS. Appl. Phys. Lett..

[69] Yin H., Gao J., Zheng G.-P., Wang Y., Ma Y. (2017). Giant Piezoelectric Effects in Monolayer Group-V Binary Compounds with Honeycomb Phases: A First-Principles Prediction. J. Phys. Chem. C.

[70] Zhuang X., He B., Javvaji B., Park H.S. (2019). Intrinsic bending flexoelectric constants in two-dimensional materials. Phys. Rev. B.

[71] Wu W., Wang L., Li Y., Zhang F., Lin L., Niu S., Chenet D., Zhang X., Hao Y., Heinz T.F. (2014). Piezoelectricity of single-atomic-layer MoS2 for energy conversion and piezotronics. Nature.

[72] Lee J.-H., Park J.Y., Cho E.B., Kim T.Y., Han S.A., Liu Y., Kim S.K., Roh C.J., Yoon H.-J., Ryu H. (2017). Reliable Piezoelectricity in Bilayer WSe_2_ for Piezoelectric Nanogenerators. Adv. Mater..

[73] Zhao M.-H., Wang Z.-L., Mao S.X. (2004). Piezoelectric Characterization of Individual Zinc Oxide Nanobelt Probed by Piezoresponse Force Microscope. Nano Lett..

[74] Wang L., Liu S., Gao G., Pang Y., Yin X., Feng X., Zhu L., Bai Y., Chen L., Xiao T. (2018). Ultrathin Piezotronic Transistors with 2 nm Channel Lengths. ACS Nano.

[75] Wang L., Liu S., Zhang Z., Feng X., Zhu L., Guo H., Ding W., Chen L., Qin Y., Wang Z.L. (2019). 2D piezotronics in atomically thin zinc oxide sheets: Interfacing gating and channel width gating. Nano Energy.

[76] Ghasemian M.B., Zavabeti A., Abbasi R., Kumar P.V., Syed N., Yao Y., Tang J., Wang Y., Elbourne A., Han J. (2020). Ultra-thin lead oxide piezoelectric layers for reduced environmental contamination using a liquid metal-based process. J. Mater. Chem. A.

[77] Li J., Zhao T., He C., Zhang K.-W. (2018). Surface oxidation: An effective way to induce piezoelectricity in 2D black phosphorus. J. Phys. D Appl. Phys..

[78] Wang X., He X., Zhu H., Sun L., Fu W., Wang X., Hoong L.C., Wang H., Zeng Q., Zhao W. (2016). Subatomic deformation driven by vertical piezoelectricity from CdS ultrathin films. Sci. Adv..

[79] Cao V.A., Kim M., Hu W., Lee S., Youn S., Chang J., Chang H.S., Nah J. (2021). Enhanced Piezoelectric Output Performance of the SnS_2_/SnS Heterostructure Thin-Film Piezoelectric Nanogenerator Realized by Atomic Layer Deposition. ACS Nano.

[80] Wang Y., Vu L.-M., Lu T., Xu C., Liu Y., Ou J.Z., Li Y. (2020). Piezoelectric Responses of Mechanically Exfoliated Two-Dimensional SnS_2_ Nanosheets. ACS Appl. Mater. Interfaces.

[81] Yang P.-K., Chou S.-A., Hsu C.-H., Mathew R.J., Chiang K.-H., Yang J.-Y., Chen Y.-T. (2020). Tin disulfide piezoelectric nanogenerators for biomechanical energy harvesting and intelligent human-robot interface applications. Nano Energy.

[82] Xue F., Zhang J., Hu W., Hsu W.-T., Han A., Leung S.-F., Huang J.-K., Wan Y., Liu S., Zhang J. (2018). Multidirection Piezoelectricity in Mono- and Multilayered Hexagonal α-In_2_Se_3_. ACS Nano.

[83] Song G., Li D., Zhou H., Zhang C., Li Z., Li G., Zhang B., Huang X., Gao B. (2021). Intrinsic room-temperature ferromagnetic semiconductor InCrTe_3_ monolayers with large magnetic anisotropy and large piezoelectricity. Appl. Phys. Lett..

[84] Dimple D., Jena N., Rawat A., Ahammed R., Mohanta M.K., De Sarkar A. (2018). Emergence of high piezoelectricity along with robust electron mobility in Janus structures in semiconducting Group IVB dichalcogenide monolayers. J. Mater. Chem. A.

[85] Xiao W.-Z., Luo H.-J., Xu L. (2020). Elasticity, piezoelectricity, and mobility in two-dimensional BiTeI from a first-principles study. J. Phys. D Appl. Phys..

[86] Tan J., Wang Y., Wang Z., He X., Liu Y., Wang B., Katsnelson M.I., Yuan S. (2019). Large out-of-plane piezoelectricity of oxygen functionalized MXenes for ultrathin piezoelectric cantilevers and diaphragms. Nano Energy.

[87] Liu S., Chen W., Liu C., Wang B., Yin H. (2021). Coexistence of large out-of-plane and in-plane piezoelectricity in 2D monolayer Li-based ternary chalcogenides LiMX2. Results Phys..

[88] Lv Q., Qiu J., Wen Q., Li D., Zhou Y., Lu G. (2023). Large in-plane and out-of-plane piezoelectricity in 2D γ-LiMX2 (M=Al, Ga and In; X=S, Se and Te) monolayers. Mater. Sci. Semicond. Process..

[89] Li Y.-Q., Zhang H.-N., Yang C., Wang X.-Y., Zhu S.-Y., Wang X.-C. (2023). Ferroelastic Zr2P2XY (X/Y = I, Br, Cl or F; X ≠ Y) monolayers with tunable in-plane electronic anisotropy and remarkable out-of-plane piezoelectricity. Appl. Surf. Sci..

[90] Qi Y., McAlpine M.C. (2010). Nanotechnology-enabled flexible and biocompatible energy harvesting. Energy Environ. Sci..

[91] Park K.-I., Xu S., Liu Y., Hwang G.-T., Kang S.-J.L., Wang Z.L., Lee K.J. (2010). Piezoelectric BaTiO_3_ Thin Film Nanogenerator on Plastic Substrates. Nano Lett..

[92] Fu Y., Luo J., Nguyen N., Walton A., Flewitt A., Zu X., Li Y., McHale G., Matthews A., Iborra E. (2017). Advances in piezoelectric thin films for acoustic biosensors, acoustofluidics and lab-on-chip applications. Prog. Mater. Sci..

[93] Wan C., Bowen C.R. (2017). Multiscale-structuring of polyvinylidene fluoride for energy harvesting: The impact of molecular-, micro- and macro-structure. J. Mater. Chem. A.

[94] Zhu G., Wang A.C., Liu Y., Zhou Y., Wang Z.L. (2012). Functional Electrical Stimulation by Nanogenerator with 58 V Output Voltage. Nano Lett..

[95] Tang G., Yang B., Liu J.-Q., Xu B., Zhu H.-Y., Yang C.-S. (2014). Development of high performance piezoelectric d33 mode MEMS vibration energy harvester based on PMN-PT single crystal thick film. Sens. Actuators A Phys..

[96] Li F., Lin D., Chen Z., Cheng Z., Wang J., Li C., Xu Z., Huang Q., Liao X., Chen L.-Q. (2018). Ultrahigh piezoelectricity in ferroelectric ceramics by design. Nat. Mater..

[97] Hwang G.-T., Annapureddy V., Han J.H., Joe D.J., Baek C., Park D.Y., Kim D.H., Park J.H., Jeong C.K., Park K.-I. (2016). Self-Powered Wireless Sensor Node Enabled by an Aerosol-Deposited PZT Flexible Energy Harvester. Adv. Energy Mater..

[98] Stoppel F., Schröder C., Senger F., Wagner B., Benecke W. (2011). AlN-based piezoelectric micropower generator for low ambient vibration energy harvesting. Procedia Eng..

[99] Doll J.C., Petzold B.C., Ninan B., Mullapudi R., Pruitt B.L. (2010). Aluminum nitride on titanium for CMOS compatible piezoelectric transducers. J. Micromech. Microeng..

[100] Persano L., Dagdeviren C., Su Y., Zhang Y., Girardo S., Pisignano D., Huang Y., Rogers J.A. (2013). High performance piezoelectric devices based on aligned arrays of nanofibers of poly(vinylidenefluoride-co-trifluoroethylene). Nat. Commun..

[101] Nan Y., Tan D., Zhao J., Willatzen M., Wang Z.L. (2020). Shape- and size dependent piezoelectric properties of monolayer hexagonal boron nitride nanosheets. Nanoscale Adv..

[102] Ma W., Lu J., Wan B., Peng D., Xu Q., Hu G., Peng Y., Pan C., Wang Z.L. (2020). Piezoelectricity in Multilayer Black Phosphorus for Piezotronics and Nanogenerators. Adv. Mater..

[103] Ding Z.-K., Zeng Y.-J., Pan H., Luo N., Zeng J., Tang L.-M., Chen K.-Q. (2022). Edge states of topological acoustic phonons in graphene zigzag nanoribbons. Phys. Rev. B.

[104] Zeng Y.-J., Feng Y.-X., Tang L.-M., Chen K.-Q. (2021). Effect of out-of-plane strain on the phonon structures and anharmonicity of twisted multilayer graphene. Appl. Phys. Lett..

[105] Zeng B., Ding Z.-K., Pan H., Luo N., Zeng J., Tang L.-M., Chen K.-Q. (2022). Strong strain-dependent phonon hydrodynamic window in bilayer graphene. Appl. Phys. Lett..

[106] Chandratre S., Sharma P. (2012). Coaxing graphene to be piezoelectric. Appl. Phys. Lett..

[107] Ong M.T., Duerloo K.-A.N., Reed E.J. (2013). The Effect of Hydrogen and Fluorine Coadsorption on the Piezoelectric Properties of Graphene. J. Phys. Chem. C.

[108] Ganatra R., Zhang Q. (2014). Few-Layer MoS_2_: A Promising Layered Semiconductor. ACS Nano.

[109] Chhowalla M., Shin H.S., Eda G., Li L.-J., Loh K.P., Zhang H. (2013). The chemistry of two-dimensional layered transition metal dichalcogenide nanosheets. Nat. Chem..

[110] Tang D., Dan M., Zhang Y. (2022). High performance piezotronic thermoelectric devices based on zigzag MoS_2_ nanoribbon. Nano Energy.

[111] Michel K.H., Verberck B. (2009). Theory of elastic and piezoelectric effects in two-dimensional hexagonal boron nitride. Phys. Rev. B.

[112] Wang D., Chen L., Wang X., Cui G., Zhang P. (2015). The effect of substrate and external strain on electronic structures of stanene film. Phys. Chem. Chem. Phys..

[113] Qiao J., Kong X., Hu Z.-X., Yang F., Ji W. (2014). High-mobility transport anisotropy and linear dichroism in few-layer black phosphorus. Nat. Commun..

[114] Ng L.-R., Chen G.-F., Lin S.-H. (2021). Generating large out-of-plane piezoelectric properties of atomically thin MoS_2_*via* defect engineering. Phys. Chem. Chem. Phys..

[115] Brennan C.J., Koul K., Lu N., Yu E.T. (2020). Out-of-plane electromechanical coupling in transition metal dichalcogenides. Appl. Phys. Lett..

[116] Cui C., Hu W.-J., Yan X., Addiego C., Gao W., Wang Y., Wang Z., Li L., Cheng Y., Li P. (2018). Intercorrelated In-Plane and Out-of-Plane Ferroelectricity in Ultrathin Two-Dimensional Layered Semiconductor In_2_Se_3_. Nano Lett..

[117] Esfahani E.N., Li T., Huang B., Xu X., Li J. (2018). Piezoelectricity of atomically thin WSe2 via laterally excited scanning probe microscopy. Nano Energy.

[118] Kang S., Jeon S., Kim S., Seol D., Yang H., Lee J., Kim Y. (2018). Tunable Out-of-Plane Piezoelectricity in Thin-Layered MoTe2 by Surface Corrugation-Mediated Flexoelectricity. ACS Appl. Mater. Interfaces.

[119] Kang S., Kim S., Jeon S., Jang W.-S., Seol D., Kim Y.-M., Lee J., Yang H., Kim Y. (2019). Atomic-scale symmetry breaking for out-of-plane piezoelectricity in two-dimensional transition metal dichalcogenides. Nano Energy.

[120] Seol D., Kim S., Jang W.-S., Jin Y., Kang S., Kim S., Won D., Lee C., Kim Y.-M., Lee J. (2021). Selective patterning of out-of-plane piezoelectricity in MoTe2 via focused ion beam. Nano Energy.

[121] Guo S.-D., Zhu Y.-T., Qin K., Ang Y.-S. (2022). Large out-of-plane piezoelectric response in ferromagnetic monolayer NiClI. Appl. Phys. Lett..

[122] Cai H., Guo Y., Gao H., Guo W. (2019). Tribo-piezoelectricity in Janus transition metal dichalcogenide bilayers: A first-principles study. Nano Energy.

[123] Zhao Y.-Z., Jia H.-J., Zhao S.-N., Wang Y.-B., Li H.-Y., Zhao Z.-L., Wu Y.-X., Wang X.-C. (2019). Janus structure derivatives SnP–InS, GeP-GaS and SiP–AlS monolayers with in-plane and out-of-plane piezoelectric performance. Phys. E Low-Dimens. Syst. Nanostruct..

[124] Hu Y., Li T., Liu L., Tan Y., Hu L., Wu K., Yang C. (2020). Janus XM-GaS (M=Si, Ge, Sn; X=N, P) monolayers: Multifunctional properties for photocatalysis, piezoelectricity and second harmonic generation. Phys. B Condens. Matter.

[125] Qiu J., Zhang F., Li H., Chen X., Zhu B., Guo H., Ding Z., Bao J., Yu J. (2021). Giant Piezoelectricity of Janus M₂SeX (M = Ge, Sn; X = S, Te) Monolayers. IEEE Electron Device Lett..

[126] Wu Y., Yang C.-H., Zhang H.-N., Zhu L.-H., Wang X.-Y., Li Y.-Q., Zhu S.-Y., Wang X.-C. (2022). The flexible Janus X2PAs (X = Si, Ge and Sn) monolayers with in-plane and out-of-plane piezoelectricity. Appl. Surf. Sci..

[127] Guo S.-D., Mu W.-Q., Xiao X.-B., Liu B.-G. (2021). Intrinsic room-temperature piezoelectric quantum anomalous hall insulator in Janus monolayer Fe_2_IX (X = Cl and Br). Nanoscale.

[128] Song G., Zhang C., Zhang Z., Li G., Li Z., Du J., Zhang B., Huang X., Gao B. (2022). Coexistence of intrinsic room-temperature ferromagnetism and piezoelectricity in monolayer BiCrX_3_ (X = S, Se, and Te). Phys. Chem. Chem. Phys..

